# A phase I clinical trial assessing the safety, tolerability, and pharmacokinetics of inhaled ethanol in humans as a potential treatment for respiratory tract infections

**DOI:** 10.3389/fmed.2024.1324686

**Published:** 2024-03-05

**Authors:** David G. Hancock, William Ditcham, Eleanor Ferguson, Yuliya V. Karpievitch, Stephen M. Stick, Grant W. Waterer, Barry S. Clements

**Affiliations:** ^1^Wal-yan Respiratory Research Centre, Telethon Kids Institute, University of Western Australia, Perth, WA, Australia; ^2^UWA Medical School, University of Western Australia, Perth, WA, Australia; ^3^School of Biomedical Sciences, University of Western Australia, Perth, WA, Australia

**Keywords:** ethanol, clinical trial, safety, tolerability, pharmacokinetics

## Abstract

**Background:**

Current treatments for respiratory infections are severely limited. Ethanol’s unique properties including antimicrobial, immunomodulatory, and surfactant-like activity make it a promising candidate treatment for respiratory infections if it can be delivered safely to the airway by inhalation. Here, we explore the safety, tolerability, and pharmacokinetics of inhaled ethanol in a phase I clinical trial.

**Methods:**

The study was conducted as a single-centre, open-label clinical trial in 18 healthy adult volunteers, six with no significant medical comorbidities, four with stable asthma, four with stable cystic fibrosis, and four active smokers. A dose-escalating design was used, with participants receiving three dosing cycles of 40, 60%, and then 80% ethanol v/v in water, 2 h apart, in a single visit. Ethanol was nebulised using a standard jet nebuliser, delivered through a novel closed-circuit reservoir system, and inhaled nasally for 10 min, then orally for 30 min. Safety assessments included adverse events and vital sign monitoring, blood alcohol concentrations, clinical examination, spirometry, electrocardiogram, and blood tests.

**Results:**

No serious adverse events were recorded. The maximum blood alcohol concentration observed was 0.011% immediately following 80% ethanol dosing. Breath alcohol concentrations were high (median 0.26%) following dosing suggesting high tissue levels were achieved. Small transient increases in heart rate, blood pressure, and blood neutrophil levels were observed, with these normalising after dosing, with no other significant safety concerns. Of 18 participants, 15 completed all dosing cycles with three not completing all cycles due to tolerability. The closed-circuit reservoir system significantly reduced fugitive aerosol loss during dosing.

**Conclusion:**

These data support the safety of inhaled ethanol at concentrations up to 80%, supporting its further investigation as a treatment for respiratory infections.

**Clinical trial registration:** identifier ACTRN12621000067875.

## 1 Introduction

The coronavirus disease 2019 (COVID-19) pandemic has highlighted the need for new therapies for both existing endemic pathogens and future pandemic preparedness (1–4). Inhalation is an under-explored option, where the major advantage is the capacity to deliver large doses directly to the target site of respiratory infection, whilst potentially minimising systemic absorption and side effects (5, 6). Ethanol aerosolised and delivered by inhalation represents a promising candidate treatment.

Whilst chronic, high-dose alcohol misuse has well-recognised detrimental effects on health, immunity, and infection outcomes, less well-recognised are the many beneficial effects from low-dose or intermittent ethanol exposures (7). In particular, the inhaled route delivering aerosolised ethanol directly to the site of infection in the upper and lower airway requires further evaluation, given consistent reports of minimal elevations in blood alcohol concentration (BAC) even after prolonged, high-dose inhalations in published human clinical (8–10), occupational (11, 12), and experimental exposure (13–15) studies. This primarily relates to ethanol’s extremely high ‘solubility’ in airway tissues, which limits uptake into the pulmonary circulation after inhalation and the risk for systemic toxicity (14–16). Instead, the ethanol preferentially concentrates in the airway mucosa and lung tissue, potentially providing topical treatment effects directly at the site of viral infection and replication. First, ethanol has direct antimicrobial activity, which can kill viruses resident in the same tissues upon contact (17, 18). Second, ethanol has potent, under-recognised, anti-inflammatory, and immunomodulatory properties with beneficial effects demonstrated in human and animal models through modulation of the core immune pathways typically dysregulated in severe infection (7, 19, 20). Finally, ethanol has surfactant-like properties, which could benefit the acute respiratory distress syndrome/pulmonary oedema seen in severe respiratory infections (8–10, 19).

Despite existing pharmacokinetic, safety, and efficacy data, inhaled ethanol has not been sufficiently explored as a viable treatment option for respiratory infections, largely due to a lack of clinical trial data. Therefore, we designed this phase I clinical trial to confirm the safety, tolerability, and pharmacokinetics of inhaled aerosolised ethanol solutions up to 80% concentration, allowing the progression to phase II trials involving infected individuals.

## 2 Methods

The study was performed by researchers from the Telethon Kids Institute at Linear Clinical Research Organisation, Nedlands, Western Australia. The study was approved by the Bellberry Ethics Committee, registered with the Australian New Zealand Clinical Trials Registry (ACTRN 12621000067875), and performed in keeping with the Declaration of Helsinki and the principles of Good Clinical Practice. All participants gave written informed consent.

### 2.1 Study design

A single-centre, phase I open-label study assessing the safety, tolerability, and pharmacokinetics of nebulised and inhaled ethanol, starting with six healthy adult volunteers without significant medical comorbidities (Group 1), followed by 12 adult volunteers with stable underlying respiratory conditions: four with stable asthma, four with stable cystic fibrosis, and four active smokers (Group 2). The group with stable respiratory conditions (Group 2) was only tested after a full Safety Monitoring Committee review of data from the six participants without significant medical comorbidities (Group 1). In both groups, a sentinel-like design was employed, where testing was temporarily halted after the first two participants in each group to allow for a thorough review of safety parameters. Upon confirming the absence of significant safety concerns in these sentinel participants, the study proceeded with the remaining subjects—four in Group 1 and 10 in Group 2.

### 2.2 Study population

Eligible participants had to be older than 18 years of age, able to give informed consent, and comply with study procedures. Healthy volunteers in Group 1 had no significant medical comorbidities based on medical history, physical examination, laboratory tests, or electrocardiogram and were non-smokers (no smoking 4 weeks prior to enrolment). Participants with underlying respiratory conditions (asthma, cystic fibrosis, or smoking) in Group 2 had stable underlying conditions as defined by no exacerbations or significant changes in treatment or smoking patterns in the preceding 4 weeks. Current smokers were defined as having smoked a minimum of 1 pack/week for 6 months. Participants in Group 2 also had no other significant medical comorbidities other than their primary condition (asthma, cystic fibrosis, or smoking). Group 2 was included to ensure ethanol’s airway pharmacokinetics were comparable in participants with and without underlying lung disease.

Participants were excluded if they were COVID-19 positive or had positive hepatitis/HIV serology. Participants were also excluded if they had chronic obstructive pulmonary disease and/or a percent-predicted forced expiratory volume in 1 s (FEV1) less than 70%, or less than 40% in those with asthma/cystic fibrosis. Participants were also excluded if they were pregnant or breastfeeding, had a known past allergy or reaction to alcohol, were unable to be exposed to alcohol for cultural/religious reasons, or had a previous history of alcohol dependence or abuse.

### 2.3 Study intervention

Medical grade ethanol solutions of 40%, 60%, and 80% concentrations v/v in water and cophenylcaine (lignocaine 5%, phenylephrine 0.5%) were supplied by GMP-certified compounding pharmacy, Optima Ovest Pharmacy, Western Australia, for aerosolisation and inhalation.

Topical analgesia was administered to participants by inhalation and topical administration 30 min before each dosing cycle to minimise nasopharyngeal discomfort. This involved inhaling nebulised cophenylcaine 4 mL through a nasal mask over 10 min, followed by the administration of three sprays (0.6 mL) into each nostril using a nasal spray bottle.

Dose-escalation involved participants performing three dosing cycles in a single visit. Cycles commenced 2 h apart, administering sequentially increasing concentrations of 40%, 60%, and 80% ethanol with each cycle. Progression to the next cycle only occurred if the previous cycle was safely tolerated, and the participant was willing to continue.

At each dosing cycle, ethanol was inhaled first nasally through a nasal mask (5 mL over 10 min) and then orally using a mouthpiece (15 mL over 30 min), with a change-over period of up to 5 min in between. Exposure to nebulisation continued for the stipulated time period or until the nebuliser pot was empty. Both cophenylcaine and ethanol were nebulised using a Pari LC Sprint driven by a standard Pari Boy SX compressor. Combined nasal and oral inhalation was chosen to simulate ethanol’s likely clinical application in respiratory infections, which typically involve both the upper and lower airways. Nasal inhalation predominately results in nasopharyngeal deposition, whilst oral inhalation results in proportionally higher lower airway deposition, so the combined protocol may better target both sites during infection.

A novel, closed-circuit reservoir spacer system (Inspiring Holdings Pty Ltd., Perth, Australia) designed specifically to prevent fugitive aerosol loss into the environment was selected and assessed in this study. This design was specifically chosen to address the known increased spread of infection by fugitive aerosol in an infectious setting. The system, which incorporates a collapsible spacer reservoir (collapsing and re-expanding with each inhalation/exhalation breath cycle), was attached to the outlet of the LC Sprint nebuliser, together with a 0.9-m extension respiratory tubing exiting the reservoir and leading to bivalved t-piece with the mouthpiece on one arm and a viral filter fitted to the exhalation arm (Supplementary Figure 1).

### 2.4 Study assessments

Primary safety outcome endpoints included blood alcohol concentrations (BACs) above 0.02%, frequency and severity of adverse events, change from baseline in vital signs (heart rate, respiratory rate, oxygen saturation, blood pressure, and temperature), clinical examination (chest auscultation, finger-nose coordination, heal-toe walking, speech assessment, subjective assessment of intoxication), electrocardiogram, laboratory tests (full blood count and liver function tests), and spirometry. Spirometry was performed using an Easy on-PC spirometer following the 2012 Global Lung Initiative (GLI) reference values. Environmental ethanol readings were measured throughout each dosing using a Tiger handheld, infrared, volatile organic compound gas detector from ION Science and placed within 1 m of the participant’s face.

Pharmacokinetic (PK) assessment analysed the relationship between breath and blood alcohol measurements. Blood alcohol concentration (BACs) measurements were performed by pathology provider PathWest, with a limit of detection of 0.001% and a laboratory error margin of 0.003%. Serial breath alcohol concentrations (BrACs) were measured using a Drager Alcotest Breathalyser (Alcolizer Pty Ltd., Perth, Australia) immediately following completion of dosing and then every minute until 15 min post-dose or a reading of zero was obtained. The upper limit of detection of the breathalyser was 0.5%, with all values above 0.5% reported as 0.5%. The breathalyser compartmental factor (1:2,100) was not removed from the breathalyser readings. All reported BAC and BrAC levels are presented as % (equivalent to gm/dL).

### 2.5 Statistical methods

The sample size chosen for this study was not based on a statistical power calculation but was chosen to provide an adequate representation for safety and tolerability assessments.

Statistical analyses were performed in R. Descriptive statistics are provided for demographic variables by treatment group with no inferential statistics. For continuous data, summaries are presented as mean and standard deviation or median and range. For categorical data, frequency counts and percentages are reported. Differences in measured parameters as compared to baseline readings were assessed for normality and then compared using either the paired *t*-tests or the Wilcoxon signed-rank tests as appropriate to their distribution.

## 3 Results

### 3.1 Demographics

Participant demographics are presented in Table 1. Participants included a range of ages (18 to 66 years), sex (6 women and 12 men), race (13 white, 3 Asian, 1 Hispanic, 1 European/Asian), and baseline alcohol consumption (0 to 18 standard drinks per week; Table 1).

**Table 1:** Baseline demographics.

	Group 1	Group 2		
	Healthy volunteers (*n* = 6)	Asthma (*n* = 4)	Smokers (*n* = 4)	Cystic fibrosis (*n* = 4)
Age (years)	34 (18–66)	28 (18–64)	44 (28–61)	38 (19–60)
Sex (*n* female/male)	3/3	2/2	0/4	1/3
**Race (*n*)**				
White	5	2	2	4
Asian	1	2	0	0
Other	0	0	2	0
Height (cm)	171 (158–180)	172 (153–185)	174 (170–185)	176 (166–181)
Weight (kg)	73 (61–88)	82 (51–93)	81 (75–112)	80 (74–91)
BMI (kg/m^2^)	25 (22–29)	26 (22–31)	27 (25–33)	27 (24–28)
Baseline FEV1 (% predicted)	98 (88–105)	90 (52–94)	86 (81–93)	61 (37–113)
Alcoholic Standard Drinks Per Week (*n*)	2 (0–10)	2 (1–10)	3 (1–18)	7 (1–9)
**Smoking status (*n*)**				
Current	0	0	4	0
Never	5	3	0	4
Former	1	1	0	0

Data are presented as median (range) or number of participants (*n*). BMI, body mass index; FEV1, forced expiratory volume in 1 s.

### 3.2 Pharmacokinetics

**Figure 1:**
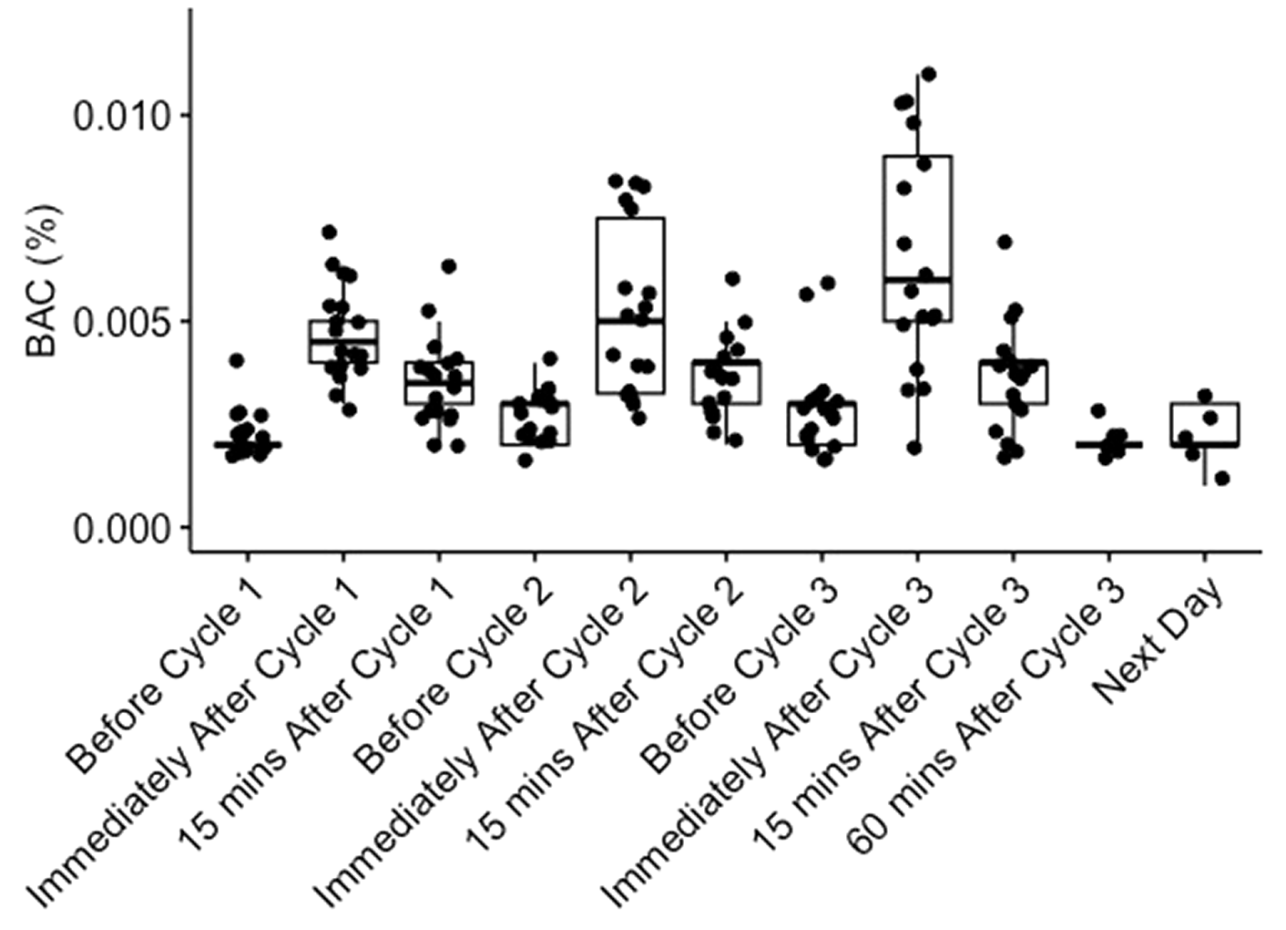
Blood alcohol concentrations (BACs) before and after dosing. BACs were measured in all participants before, immediately after, and 15 min after dosing with 40% (Cycle 1), 60% (Cycle 2), and 80% (Cycle 3) ethanol v/v in water. BACs were also measured 60 min and the day after completion of 80% dosing in a subset of individuals. Each dot represents a BAC reading from an individual participant.

Blood alcohol concentrations (BACs) were measured before and after each dosing period. Median (range) BACs immediately following 40%, 60%, and 80% dosing were 0.005% (0.003 to 0.007%), 0.005% (0.003 to 0.008%), and 0.006% (0.002 to 0.011%), respectively, decreasing to 0.004% (0.002 to 0.006%), 0.004% (0.002 to 0.006%), and 0.005% (0.002 to 0.007%), respectively, 15 min after dosing (Figure 1). When measured in a subset of participants at 1 h and the day after 80% dosing, all blood alcohol values were at pre-dosing levels (range 0.001% to 0.003%; Figure 1). No significant differences in BAC levels were noted between healthy participants without underlying comorbidities (Group 1) and participants with stable underlying respiratory conditions (asthma, cystic fibrosis, and smoking cystic fibrosis; Group 2).

**Figure 2:**
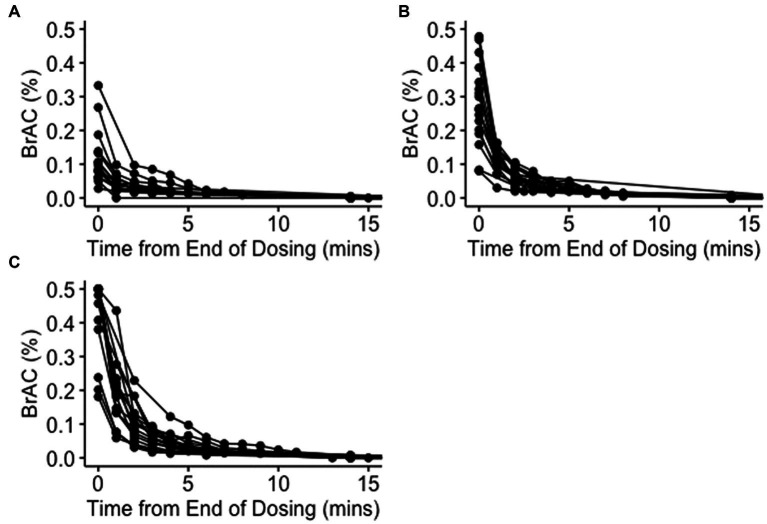
Breath alcohol concentrations after dosing. Serial breath alcohol concentrations (BrACs) were measured minutely after dosing until a value less than 0.02% was recorded and then again 15 min after dosing with 40% **(A)**, 60% **(B)**, and 80% **(C)** ethanol v/v in water. Each line represents serial BrAC readings from an individual participant.

Serial breath alcohol concentration (BrAC) readings varied significantly between participants, but the peak reading and time to a breathalyser reading of less than 0.02% correlated with the concentration of ethanol inhaled (Figure 2). The median (range) BrACs were 0.096% (0.028 to 0.333%), 0.265% (0.080 to 0.478%), and 0.5% (0.181 to 0.5%) immediately following 40, 60, and 80% dosing, respectively. Median BrACs at 15 min and 1–2 h after dosing in all cycles were 0.000%. No significant differences in BrAC readings were observed between participants without underlying comorbidities (Group 1) and participants with stable underlying respiratory conditions (Group 2).

There was a poor correlation between BAC and BrAC measurements (R^2^ = 0.23), with BrAC readings on average 50-fold higher than the corresponding BAC reading taken at the same time point.

### 3.3 Safety

No BAC greater than 0.02% were identified at any time point (Figure 1). No serious adverse events were observed, with 21 adverse events recorded amongst 14 of 18 participants (Table 2). The most frequently reported adverse events were nasopharyngeal or breathing discomfort (six participants), lightheadedness (two participants), and headache (two participants; Table 2; Supplementary Table 1). All adverse events were transient and self-resolving. No evidence of acute intoxication was observed.

**Table 2:** Adverse events by group.

	Group 1	Group 2		
	Healthy volunteers (*n* = 6)	Asthma (*n* = 4)	Smokers (*n* = 4)	Cystic fibrosis (*n* = 4)
**Adverse events, *n* (%)**				
Any	4 (66%)	2 (50%)	4 (100%)	4 (100%)
Serious	0	0	0	0
**Adverse events description, *n***				
Nasopharyngeal discomfort	1	1	1	1
Breathing discomfort		1		1
Lightheadedness	1		1	1
Headache	2			
Increased phlegm expectoration		1		
Unsteady gait	1			
Cannula site pain			1	
Chest tightness				1
Asthma exacerbation		1		
Contact dermatitis to dressing				1
Pre-syncope following venepuncture			1	

There were no significant differences in spirometry readings from pre-dosing baseline over the study period in any group. Compared with pre-dosing spirometry, the mean (range) absolute change in the FEV1 was +0.6% (−8 to +18%), +1.9% (−6 to +16%), and + 0.6% (−10 to +15%) after 40, 60, and 80% dosing, respectively. Statistically significant increases in heart rate were observed during both nasal (mean increase +4.5 bpm) and oral (mean increase +3.9 bpm) inhalation as compared to pre-dosing readings. Heart rate increases were considered not to be clinically significant and returned to pre-dosing levels after each dosing cycle. There were also statistically significant increases in systolic (mean increase +2.7 mmHg) and diastolic (mean increase +3.7 mmHg) blood pressure readings during oral inhalation. Again, these were not considered to be clinically significant and normalised on completion of dosing. No significant alterations in respiratory rate, oxygen saturation, or temperature were observed during dosing. No significant electrocardiogram abnormalities were detected.

There was no statistically significant change in liver function tests (enzyme and bilirubin levels) both 1 h and the day after completion of all dosing cycles when compared to the pre-dosing baseline. There was a statistically significant but transient increase in neutrophil count (median 3.39 pre-dose vs. 4.98 ×10^9^/L post-dose, value of p 0.027) 1 h after dosing that normalised by the next day. There was no significant change in cell counts for red blood cells, platelets, or other immune cell populations.

### 3.4 Tolerability

Of 18 participants, 15 tolerated the full study protocol with all 18 finishing 40% dosing, 17 finishing 60% dosing, and 15 finishing 80% dosing. In the cystic fibrosis group, one participant withdrew 2 min into 60% oral inhalation due to nasopharyngeal discomfort, and another withdrew 7 min into 80% oral inhalation due to breathing discomfort. Neither participant completed any further dosing. One participant in the current smoker group stopped 4 min into 80% nasal dosing due to nasal discomfort but then went on to complete 80% oral dosing.

Eleven participants reported some discomfort with nasal inhalation, whilst 14 reported some discomfort with oral inhalation. The relative tolerability of nasal vs. oral inhalation varied on an individual participant basis with some participants finding nasal inhalation less comfortable and vice versa. However, 17 of 18 participants responded with ‘Agree’ or ‘Strongly Agree’ to repeating the treatment if medically required.

### 3.5 Closed-circuit delivery system

Measurement of ambient environmental fugitive ethanol escape was performed throughout dosing. Only a small increase in mean environmental ethanol readings was recorded during dosing (mean (SD) 7.7 (0.04) ppm) as compared to average readings taken before the start of dosing (mean 6.6 (0.03) ppm). No device issues were noted, and feedback on usability was highly supportive of the protection offered by the inhalation spacer system.

## 4 Discussion

In this study, inhaling ethanol aerosol continuously for 10 min nasally and 30 min orally, resulted in only minimal elevations in BAC. Even at the highest concentration (80% ethanol v/v in water), a median BAC reading of 0.006% (0.002%–0.011%) was observed immediately following dosing (Figure 1). These BACs are five-fold lower than what would be expected for an average adult consuming a similar volume of 80% ethanol orally over a 30-min period (~0.02–0.03%), and well below the level where the symptoms of intoxication might be expected to occur.

The observed BACs are in keeping with those reported in previously published studies (8–15) and fit with ethanol’s known high air-tissue partition coefficient, tissue uptake, and clearance kinetics, well-demonstrated in seminal studies (14–16). When the human airway is exposed to high concentrations of ethanol aerosol or vapour, the partition coefficient results in preferential uptake into lung tissue with the rate dictated by the concentration gradient. For comparison, oxygen and anaesthetic gases typically used in operating theatres are relatively ‘insoluble’ in airway tissues (1,800-fold lower air-tissue partition coefficient) and travel all the way to the alveoli before freely diffusing into the pulmonary circulation. In contrast, ethanol preferentially diffuses into the lung tissue which, during respiratory infection, is usually the main site of viral replication and inflammation. In the lung tissue, ethanol is absorbed into the capillary bed of the bronchial circulation, although this accounts for only 1%–2% of the circulating volume of the pulmonary circulation. The lower-than-anticipated systemic uptake of ethanol inhaled into the airways is readily explained. The concentration gradient in the airway is reversed on cessation of nebulised aerosol inhalation, causing ethanol in the tissues to readily diffuse back into the airway lumen and be cleared on successive exhalations (14–16).

The high breathalyser readings obtained at the end of each dosing and their lack of any notable correlation with contemporaneous blood levels (mean BrAC of 0.4% and BAC of 0.006% immediately after 80% dosing) appear to correlate with a significant uptake of ethanol by the lung tissue in the concentration-dependent manner described (Figures 1, 2). Although adequate tissue levels are an important goal if ethanol’s postulated topical treatment effects are to be exploited, the optimal levels for efficacy are unknown. Unfortunately, the breathalyser machines used in this study were only capable of measuring a maximum BrAC of 0.5%, which was exceeded in many participants. Breathalysers capable of measuring higher maximum levels or continuous mass spectrometry measurements of exhaled breath ethanol could be used to better quantify achieved tissue concentrations. The high degree of variability in breathalyser measurements of peak exhaled concentration (BrAC range 0.028–0.5%; Figure 2) could be attributed to several factors, including participant-specific characteristics such as breathing depth and rate during or after dosing, resulting in relatively higher or lower dose delivery and/or relatively faster or slower clearance. Observation supported participants who experienced less discomfort and were able to breathe normally during ethanol inhalation, demonstrated higher tissue levels. Other factors likely to influence the level and variability of airway ethanol concentration include disease state, lung surface area, ventilation/perfusion dynamics, age, and airway surface liquid and mucous volumes.

Measurement of the effects of ethanol dosing on other safety parameters revealed no clinically significant alterations in electrocardiogram recordings, spirometry, respiratory rate, oxygen saturation, temperature, and most blood markers. Ethanol has well-defined bronchodilator properties, and in one asthmatic participant, FEV1 increased by 10% following inhalation of 40% dosing, and this was sustained through 60% and 80% dosings. Mild, albeit statistically significant, elevations in heart rate and blood pressure that normalised on cessation of dosing were observed. Given that these events appeared to occur more frequently in participants reporting significant discomfort on inhalation and occurred in the absence of significantly elevated BACs, these elevations were more likely due to stress/discomfort (21). As raised blood ethanol levels are normally associated with a reduction in blood neutrophil counts, the observed elevations in neutrophil counts following dosing (that normalised by the next morning) could reflect a stress response (22). Three participants were unable to complete dosing due to their inability to tolerate the procedure. Although these participants’ safety data were no different than the other participants, this level of discomfort may limit the acceptability of inhaled ethanol for some individuals as a regular treatment, particularly at high concentrations.

Whilst efficacy assessments have been limited by concerns around the recognised side effects associated with alcohol abuse, there is sufficient existing data to support ethanol’s beneficial effects in human respiratory disease. Ethanol was used as a mainstay of treatment in cardiogenic pulmonary oedema in the 1950s–1970s, with significant clinical efficacy and no major side effects or elevations in blood alcohol concentration reported (8–10). A combined ethanol and dimethyl sulphoxide nasal spray has recently shown promise in protecting against COVID-19 infection when used as prophylaxis in at-risk healthcare workers (17). Finally, a Japanese case report reported a rapid improvement in oxygenation and reduction in inflammatory markers (neutrophil elastase, interleukin-8, and surfactant protein-D) in a patient presenting with acute respiratory distress syndrome after they were treated with two doses of ethanol instilled via an endotracheal tube (19). Outside of human studies, inhaled ethanol has also been shown in mouse models to protect against lethal influenza infection when delivered prophylactically (20). In contrast to these beneficial effects reported with topical airway administration via inhalation, heavy, prolonged oral alcohol misuse has been typically associated with immune suppression and worse infection outcomes (7, 23). Collectively, these data suggest that the route of administration, dose, and duration of exposure play critical roles in modulating ethanol’s potential beneficial or negative effects on the human body.

Whenever ethanol is considered a medication, an important consideration is its potential for abuse. Although inhalation is unlikely to become a primary method for alcohol misuse due to tolerability issues, the requirement for specialised equipment, and the inability to generate high blood concentrations, there remains an unknown risk of precipitating addiction responses from the small (but measurable) increase in BACs and smell/taste sensations during inhalation.

For this study, inhaled ethanol was delivered through a novel closed-circuit reservoir spacer system aimed at improving both the safety and efficiency of drug delivery. This closed-circuit system’s capacity to minimise fugitive aerosol loss during highly volatile ethanol nebulisation was confirmed by only negligible increases in environmental ethanol levels, with measured levels (typically 0–10 ppm) consistent with those seen in hospital settings with ethanol-based hand sanitiser use (24). The low levels of environmental ethanol detected in this study support further research into closed-circuit nebuliser systems to improve safety during nebulised inhaled treatment administration in infectious environments.

The measured data from this study were reassuring in that they were consistent with previous studies assessing ethanol inhalation, as well as being consistent across healthy participants and participants with a range of common respiratory conditions. As the study assessed an uninfected population, safety would need to be confirmed in infected persons and/or persons with unstable respiratory diseases. In this study, the open-label design was unavoidable due to the unique smell, taste, and sensation of ethanol during inhalation, and this will be the same for future phase II efficacy studies.

In conclusion, although some tolerability issues were observed, this study confirmed that inhaled aerosolised ethanol in high concentrations is safe and results in high tissue concentrations but low blood concentrations, with a highest BAC of 0.011%. These observations, together with abundant pre-existing safety and efficacy data, support further evaluation in phase II clinical trials assessing the safety and efficacy of inhaled ethanol as a treatment for patients with respiratory infections.

## Data availability statement

The original contributions presented in the study are included in the article/Supplementary material, further inquiries can be directed to the corresponding authors.

## Ethics statement

The studies involving humans were approved by Bellberry Human Research Ethics Committee. The studies were conducted in accordance with the local legislation and institutional requirements. The participants provided their written informed consent to participate in this study.

## Author contributions

DH: Conceptualization, Formal Analysis, Investigation, Visualization, Writing – original draft. WD: Conceptualization, Investigation, Methodology, Supervision, Writing – review & editing. EF: Investigation, Methodology, Writing – review & editing. YK: Visualization, Writing – review & editing. SS: Conceptualization, Supervision, Writing – review & editing. GW: Conceptualization, Supervision, Writing – review & editing. BC: Conceptualization, Funding acquisition, Investigation, Supervision, Validation, Writing – original draft.
