# Supplementary material for: A phase I clinical trial assessing the safety, tolerability, and pharmacokinetics of inhaled ethanol in humans as a potential treatment for respiratory tract infections
Source: Front Med (Lausanne). 2024 Mar 5;11:1324686. doi: 10.3389/fmed.2024.1324686 (PMC10949138; doi:10.3389/fmed.2024.1324686)
Supplement: Supplementary file 1 [file Data_Sheet_1.docx]

**A Phase I Clinical Trial assessing the Safety, Tolerability, and Pharmacokinetics of Inhaled Ethanol in Humans as a Potential Treatment for Respiratory Tract Infections**

David G Hancock^1,2*^, William Ditcham^1^, Eleanor Ferguson^1^, Yuliya V Karpievitch^1^, Stephen M Stick^1,2^, Grant W Waterer^2^, Barry S Clements^1,2*^

**SUPPLEMENTARY INFORMATION**

**
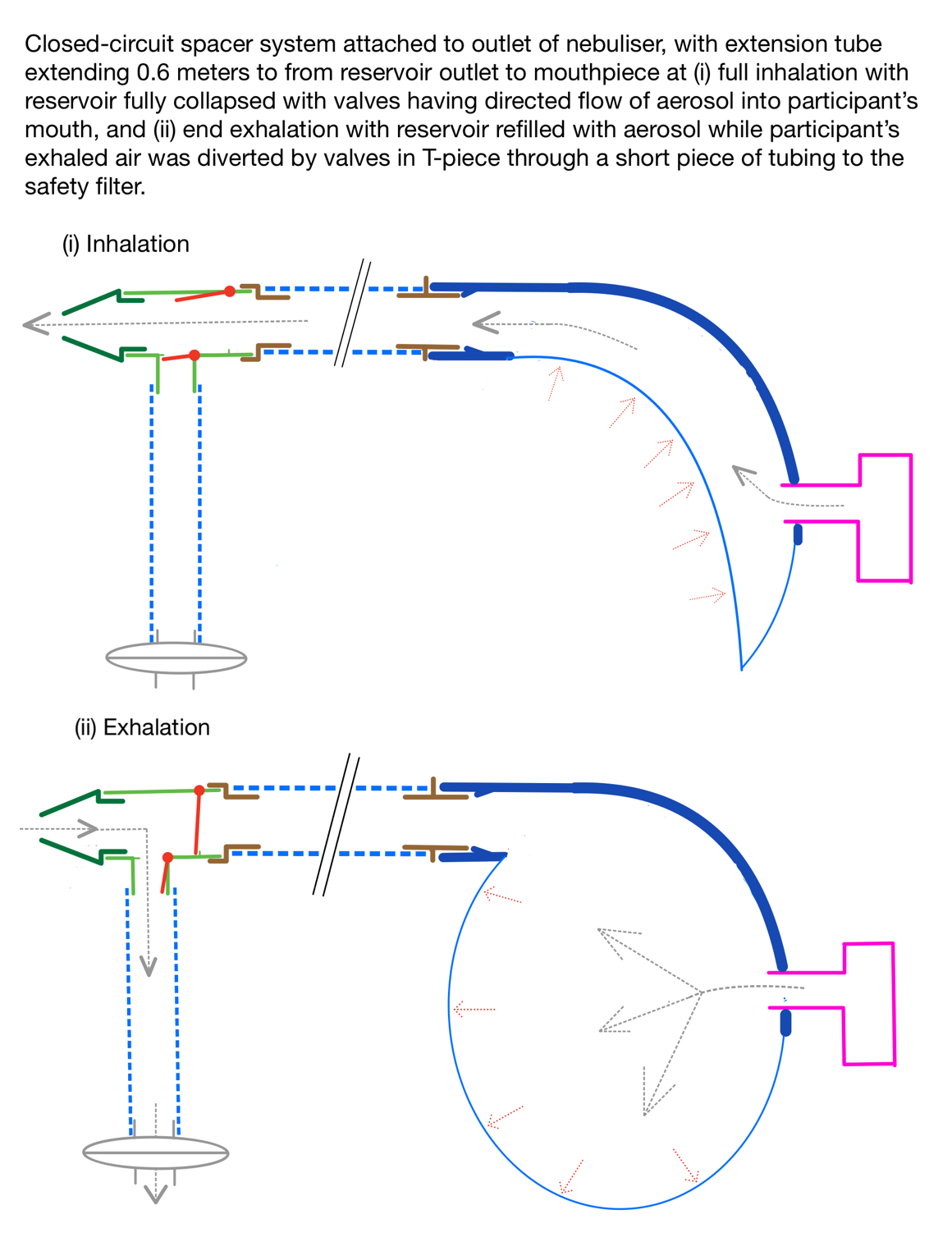
**

**Supplementary Figure 1.** Schema of novel, closed-circuit, reservoir spacer system

| **Adverse Event** | **Group** | **Relationship: to Drug or Nebulisation** | **CTCTAE** | **Timing Relative to Dosing Cycle** |
| --- | --- | --- | --- | --- |
| Breathing Discomfort | 2: Cystic Fibrosis | Related to drug | 3 | During Cycle 3 dosing |
| Breathing Discomfort | 2: Asthma | Related to drug | 1 | During Cycle 3 Dosing |
| Cannula site pain | 2: Smoker | Not Related | 1 | Evening after all dosing |
| Chest Tightness | 2: Cystic Fibrosis | Not Related | 1 | 4 days after dosing |
| Contact dermatitis to  cannula dressing | 2: Cystic Fibrosis | Not Related | 1 | Evening after all dosing |
| Exacerbation of asthma - likely infection | 2: Asthma | Not Related | 1 | 3 days after dosing |
| Headache | 1: Healthy | Not Related | 1 | Evening after all dosing |
| Headache | 1: Healthy | Not Related | 1 | During Cycle 2 dosing |
| Headache | 1: Healthy | Not Related | 1 | During Cycle 3 dosing |
| Lightheadedness | 1: Healthy | Related to nebulisation | 1 | During Cycle 1 dosing |
| Lightheadedness | 2: Smoker | Related to nebulisation | 1 | After Cycle 1 dosing |
| Lightheadedness | 2: Cystic Fibrosis | Related to nebulisation | 1 | During Cycle 1 dosing |
| Nasopharyngeal  discomfort | 1: Healthy | Related to drug | 1 | During Cycle 2 dosing |
| Nasopharyngeal  discomfort | 2: Smoker | Related to drug | 1 | During Cycle 3 dosing |
| Nasopharyngeal  discomfort | 2: Cystic Fibrosis | Related to drug | 3 | During Cycle 2 dosing |
| Nasopharyngeal  discomfort | 2: Asthma | Related to drug | 1 | During Cycle 3 dosing |
| Phlegm expectoration | 2: Asthma | Related to drug | 1 | Day after all dosing |
| Pre-syncope following  venepuncture | 2: Smoker | Not Related | 1 | Day after all dosing |
| Unsteady gait | 1: Healthy | Related to nebulisation | 1 | After Cycle 1 dosing |

**Supplementary Table 1. Detailed Adverse Events**
